# Correlation: Between Autochthonous Microbial Diversity and Volatile Metabolites During the Fermentation of Nongxiang Daqu

**DOI:** 10.3389/fmicb.2021.688981

**Published:** 2021-09-22

**Authors:** Yuke Deng, Dan Huang, Baolin Han, Xinqian Ning, Dong Yu, Huixiang Guo, Yufang Zou, Wen Jing, Huibo Luo

**Affiliations:** ^1^College of Bioengineering, Sichuan University of Science and Engineering, Zigong, China; ^2^Key Laboratory of Brewing Biotechnology and Application, Sichuan Province, Sichuan University of Science and Engineering, Zigong, China; ^3^Sichuan Tuopai Shede Liquor Co., Ltd., Suining, Sichuan

**Keywords:** nongxiang daqu, microbial communities, differential metabolites, process of fermentation, correlation analysis

## Abstract

Daqu is an important saccharifying and fermenting agent. It provides various microorganisms and enzymes for the fermentation of Baijiu and plays a vital role in the formation of Baijiu flavor. However, it is difficult to obtain information on microbial growth and metabolism in time for Daqu production. Therefore, the “Qu Xiang” obtained by smelling is an important index in the traditional production process to evaluate the microbial fermentation in the process of Daqu-making, “Qu Xiang” mainly represents the volatile flavor compounds in Daqu. The microbial diversity and volatile metabolites on 0, 6, 16, and 29 days of the fermentation process were measured using high-throughput sequencing and gas chromatography–mass spectrometry. Significant differences were found in the composition of the microbial community. *Pseudomonas*, *Weissella*, *Bacillus*, and *Pelomonas* were the main bacterial genera. *Alternaria*, *Rhizopus*, and *Pichia* are the main fungal genera. A total of 32 differential volatile metabolites were detected in samples at four time points using differential metabolic analysis. The correspondence of prevailing microorganisms with differential metabolites distinguished by Spearman correlation and two-way orthogonal partial least square analysis show that Saccharopolyspora exhibited a significant connection for the 12 differential metabolites. A significant positive correlation was observed between *Rhizomucor* and 13 different metabolites. These findings further understanding of the metabolism of microorganisms in Daqu fermentation and also help to control the microorganisms in the Daqu-making process, to obtain more stable Baijiu products.

## Introduction

Fermentation is a traditional strategy that has been used to process and preserve foods all over the world. It has significance in cultural heritage and provides local economic products and rich microbiological resources (Liu and Tong, 2017). Chinese baijiu is a kind of prevalent beverage that is enjoyed around the world. Nongxiang baijiu is the most famous type and has a long history. The annual output accounts for more than 70% of Chinese liquor as a result of its unique flavor and aroma (Zou et al., 2018). Nongxiang Baijiu is brewed by solid-state fermentation, in which the production of Daqu and grain fermentation is mainly involved. Its characteristics are acquired from Daqu, a traditional fermentation starter (Du et al., 2019). Daqu is produced from raw materials in an open environment without sterilizing. During this period process, liquefaction enzymes, saccharifying enzymes, proteases, and esterifying enzymes were accumulated through microbial metabolism (Wu et al., 2020). In Baijiu liquor, the starch and protein in the Daqu can be degraded into small molecules by the aforementioned enzymes, hence serving as nutrients and substrates for microbial growth and flavor metabolism (He et al., 2020). These enzymes are mainly produced by some specific microorganisms, such as *Aspergillus*, *Rhizopus*, *Bacillus*, *LactoBacillus*, *Wickerhamomyces*, and *Saccharomycoposis* (Liu and Sun, 2018; Li et al., 2020). The interaction between microbes, microorganisms, and the environment in the manufacturing process of Daqu making eventually forms a Daqu microbial group that is involved in the subsequent Baijiu brewing (Chen et al., 2020; Fan et al., 2020). Previous research found that the proportion of bacteria and fungi from Daqu was 81.5% and 79.2% respectively, in the fermented grain (Wang P. et al., 2017). They not only affect the accumulation of enzymes in the fermentation process but also determine the final community composition (Wang et al., 2021). Therefore, the Daqu microorganism group has a crucial effect on the quality formation of Nongxiang Baijiu. If we master the rule of microbial succession in the production process of Nongxiang Daqu, we can get high-quality Daqu.

However, it is difficult to obtain timely information on microbial growth and metabolism in time and quickly during the production process (Fan et al., 2019). Therefore, the “Qu Xiang” obtained by smelling is an important index to evaluate microbial fermentation in the process of Daqu making. As a sensory index, “Qu Xiang” mainly represents the volatile flavor compounds in Daqu. Meanwhile, metabolism is the most downstream of life activities and the change in the upstream signal can be easily reflected in the metabolic phenotype through the cascade amplification effect, so metabolism has high sensitivity as a marker (Mousavian et al., 2016; Noda-Garcia et al., 2018; Slot and Gluck-Thaler, 2019). It was reported that the volatile components of Daqu exhibit diversity. For instance, a previous study shows that hexanal, 4-guaiacol, and phenylacetaldehyde are crucial aroma-active components in Daqu, which may be related to the Daqu quality. There are multiple and diverse species inhabiting Daqu, *LactoBacillus*, *Bacillus*, *Aspergillus*, and some *Saccharomyces* genera (*saccharomycoposis*, *Wickerhamomyces*, and *Pichia*) dominate different types of Daqu (Xiao et al., 2017; Jin et al., 2019). Researchers have learned little about the change of the microbial communities that are associated with flavor formation during the production of Daqu and how these microbial communities produce flavor substances through metabolic pathways. The use of Daqu with defined standard quality is one of the most effective potential methods for standardizing fermentation and ensuring the stable quality of Nongxiang Baijiu. Therefore, the study of microbial flora changes during the Daqu making process, combining this with metabonomics analysis could characterize microbial metabolic activity by different metabolites and provide a theoretical basis for the real-time monitoring of microbial growth and metabolism in the Daqu making process.

The primary aims of the present study were as follows: (1) using a culture-independent strategy during the producing process of fermentation to explore the structures of the bacterial and fungal communities; (2) to determine the content of volatile metabolites during fermentation; (3) to reveal potential correlations between microbial communities and metabolites. Then, to prove that the change of the relative abundance of the microorganisms can be reflected by the change of metabolite content. If we achieve these objectives it would further our understanding of the relationship between microorganisms and metabolites in Daqu and provide us with a theoretical basis for the quality monitoring of Daqu fermentation.

## Materials and Methods

### Sampling

**FIGURE 1:**
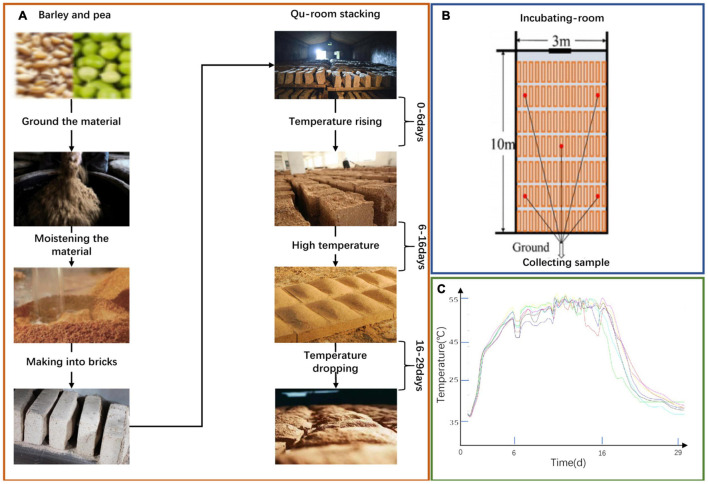
A schematic of the production process of NongXiang Daqu **(A)**. A Schematic diagram of increasing room **(B)**. Temperature curve of the fermentation process **(C)**.

We collected Daqu samples from a Nongxiang Baijiu enterprise in Suining, Sichuan, China. They were collected from five locations within the same incubating room and they were then mixed as one sample. The collection times were on days 0, 6, 16, and 29, respectively. The details of the sample are shown in Figures 1A-C. First, crushed barley and pea was mixed with water and then pressed to fall into a cuboid brick shape (Daqu bricks). Then, the bricks were placed into an incubating room, cultured for about 30 days according to the enterprise operation regulations. The culture temperature of Daqu needs to be controlled in line with the following procedure. (i) During the low-temperature period, microbiota start to grow and the temperature gradually increases, reaching 30–40°C in 2–3 days. (ii) In the high-temperature incubation stage, the temperature increases by 5–8°C/day to a maximum of 55–62°C. In this period, the relative humidity of the incubating room is increased to 90%, doors and windows are properly opened to ventilate and lower the humidity. (iii) For the temperature dropping stage, the temperature and relative humidity decrease gradually, and the aroma compounds are accumulated by the metabolic conversion. Finally, Daqu were stacked in another incubating room and deposited for more than three months until maturation. The samples were conveyed to the laboratory on ice in an insulation container. Samples were stored at −20°C before analysis of volatile compounds and were deposited at −80°C before DNA extraction. All analyses were conducted in biological quadruplicate.

### DNA Extraction and PCR Amplification Genomic

A 2 g portion of each of the crushed *Daqu* samples was ground into much tinier particles using a Medium-Flux Tissue Grinding System (Dinghaoyuan, China) combined with freezing in liquid nitrogen for homogenization. We used the Soil DNA Kit (Omega Bio-Tek, Norcross, GA, United States) to extract the DNA from the microbial communities of these samples. The DNA extract was tested for quality on 1% agarose gel, the concentration and purity of DNA were identified by using the Nano Drop 2000 UV–vis spectrophotometer (Thermo Scientific, DE, United States). The V3-V4 regions of the bacterial 16S rRNA gene and the internal transcribed spacer (ITS1) regions of fungal rRNA genes were amplified using the primers 338f/806r (5′-ACTCCTACGGGAGGCAGCAG-30 /50- GGACTACHVGGGTWTCTAAT-3′) and ITS1f/2043R (5′-CTTGGTCATTTAGAGGAAGTAA-3′/5′-GCTGCGTTCTTCATC GATGC-3′) with barcodes, respectively (Zhao et al., 2016). The polymerase chain reaction (PCR) amplification of the gene was performed and the initial denaturation was conducted at 95°C for 3 min, followed by 27 cycles of denaturation at 95°C for 30 s; annealing at 55°C for 30 s and extension at 72°C or 45 s; single extension at 72°C for 10 min; and ultimately at 4°C. The PCR mixtures contained the following: 4 μL of 5 × Trans Start Fastpfu buffer, 2 μL of 2.5 mM dNTPs, 0.8 μL of the forward primer (5 μM), 0.8 μL of the reverse primer (5 μM), 0.4 μL of Trans Start FastPfu DNA polymerase, 10 ng DNA of the template, and up to 20 μL of ddH_2_O.

### Pyrosequencing and Bioinformatics Processing

Illumina MiSeq platform was used to carry out two terminal (Paired-end) sequencing of DNA fragments from the community. To integrate the original two terminal sequencing data, the quality of the two terminal sequences in fastq format was screened one by one by using the sliding window method. The size of the window was 10 bp, the step size was 1 BP, and the average quality of the base in the window was required to be ≥Q20 (that is, the average sequencing accuracy of the base was ≥99%). The length of the truncated sequence should be more than 150 BP, the existence of (mbiguous base) N is not allowed. Then, using Flash software (v1.2.7^1^), pairing the two terminal sequences that passed the quality screening according to the overlapping base. The length of the overlapping base of read 1 and read 2 is required to be more than 10 bp, and the number of base mismatches is less than 10% of the length of the overlapping base. Finally, according to the index information of each sample (barcode sequence, which is a small base sequence used to identify the sample at the beginning of the sequence), the connected sequence recognition is assigned to the corresponding sample (index sequence is required to match completely), to obtain the effective sequence of each sample. Operational taxonomic units (OTUs) with 97% similarity cutoffs were clustered using UPARSE (version 7.1) (Edgar, 2013), chimeric sequences were identified and removed using the UCHIME algorithm. Taxonomic assignment was conducted using RDP Classifier (Wang et al., 2007). Alpha diversity indices Chao1, Shannon, and Goods coverage were performed in the software package mothur to reflect the diversity and richness of microbial communities in different samples (Schloss et al., 2011).

### Quantitative Real-Time PCR

The qPCR reagent used was AceQ^®^ qPCR SYBR^®^ Green Master Mix of Applied Biosystems^®^ by Vazyme (Carlsbad, CA, United States) and a final reaction volume of 20 μL was used. qPCR and melt curve analysis were performed on an Applied Biosystems^®^7500 Real-Time PCR System, Software v.2.0 (Foster City, CA, United States).1 ul of DNA was diluted several times and used as a template. The system includes an 8ul template and 8ul mixture of 2 × SYBR real-time PCR premixture and general primers what was mentioned in the previous PCR method. The qPCR protocol began with 1 cycle of 95°C for 5 mins, followed by 40 cycles of 95 C for 15 s, 60 C for 30 s was executed for 40 cycles. The melting curve stage (60–95°C) was added after the step of amplification.

### Determination of Organic Acids in Daqu

Put a 1 g sample into a test tube, then add 10 ml ultra pure water. After ultrasonic treatment in a water bath for 30 min, centrifugation at 5000 rpm for 10 min, and filtering it through 0.22 μm PTFE membrane (allcrom, Sao Paulo, Brazil). A high-performance liquid chromatography system (LC- 20AT; Shimadzu, Kyoto, Japan) was used to analyze organic acids (malic acid, citric acid, oxalic acid, organic acid, succinic acid, acetic acid, acetic acid, and tartaric acid); Oven for controlling column temperature, UV relative detector; and a data acquisition system fitted with a CAPECELL PAK MG S5 C18 column (4.6 × 250 mm 2, 5 μm). The mobile phase consists of a 0.01M potassium dihydrogen phosphate buffer (ph2.55) and methanol (90:10, v/v) solution. The autosampler was adjusted to 20 μL. The isocratic elution method was applied to the mobile phase with a flow rate of 1.0 ml/min. The wavelength of 210 nm was selected for quantization. The calibration curves of different compounds were constructed by using pure standard curves of different concentrations (de Sena Aquino et al., 2015).

### Determination of Volatile Metabolites

Gas chromatography-mass spectrometry (GC-MS) was used. Headspace solid phase microextraction (HS-SPME) combined with GC-MS (7890bgc, 5977ams) was used to analyze the volatile metabolites of fermented Daqu (Agilent Technologies, Santa Clara, United States). The sample (1 g) was accurately weighed into a 20 ml glass bottle containing 3ml saturated salt water, and then 50 μL 2 octanol (10 – 6 mol/L) was used as internal standard. The vials were immediately put into a heating block and equilibrated at 70c for 15 min, then DVB/car/pdmsspme fiber (2 cm, 50/30 μm) (Supelco, PA, United States) was exposed to the top of the samples for 40 min at 70°C. In the split free mode, the ejector removes the volatiles from the SPME fiber at 250°C for 5 min. Helium was used as carrier gas at a flow rate of 1.0 ml / min. Db-5ms column (30M × two hundred and fifty μm,0.25 μ M film thickness; Agilent, Santa Clara, United States) with column temperature set to 40°C (keep for 3 min) was increased to 150°C at 5°C /min, then 250°C at 10°C / min (keep at 10 min). MS was carried out at 230°C, M / Z 33-500 and 70 ev. The average concentration of each compound was calculated using the following formula: odorant = concentration (internal standard of the compound) × Peak area concentration) internal standard peak area.

### Statistical Analysis

Data were analyzed using statistical products and services solutions (version 17.0; SPSS software in Chicago, IL, United States) and Duncan’s test. Differential metabolites were identified by using SIMCA-P software (version 11.5; Umetrics, Umeå, Sweden). To analyze the differences in volatile profiles among the samples; variables with a VIP value of 2 and a Student’s *t* test *p* value of 0.05 were considered differential. The quality of the OPLS-DA models was verified by the typical cross-validation procedure of leaving one seventh of the samples out of each round and then assessing the model. Data drawing also used the GraphPad prism (version 7; GraphPad Software, San Diego, CA, United States) and OriginPro (version 7.5; OriginLab Corporation, Northampton, United States) for plotting data. Correlation analysis was performed using R (version 3.2.2; The R Foundation for Statistical Computing, Vienna, Austria). The Phylogenetic Investigation of Communities by Reconstruction of Unobserved States (PICRUSt2) analysis was performed based on the 16S rRNA sequencing to gain additional insight into the metabolic potential of the differential fermentation period in Daqu. The gene family counts for each predicted sample were derived from the Kyoto Encyclopedia of Genes and Genome (KEGG) ortholog (KO)^2^. The estimated KEGG pathways related to the production of volatile metabolites were manually classified based on their KEGG identification (Yang et al., 2019). Statistically significant differences were accepted at *p* < 0.05. Spearman correlations coefficient (*r*) was calculated among the microbial genera and metabolites to detect the association between the microbiota and volatile compounds, *r* > 0.6 with statistical significance was considered as a strong correlation. Using Cytoscape 3.4.0 to visualize the correlation matrix.

## Results

### Quantitative Real-Time PCR *Analysis*

We operated qPCR for bacteria and fungi in the samples respectively and drew the melting curve (Supplementary Figure 1). During Daqu fermentation, the copy numbers of bacteria and fungi showed an obvious upward trend. The bacterial copy number ranged from 4262500 to 16401415, and the fungal copy number ranged from 21625000 to 225075000. The melting curve showed only one peak, which proved that the reaction was specific and there was no dimer.

### Diversity and Composition of Microbial Community

The numbers of effective sequences of the fourteen samples were in the range of 37,082–100,172 and 27,082–91,208 for bacteria and fungi, respectively. The average number and ratio of high-quality sequences were 26818 and 85.97% for bacteria, and 60641 and 96.85% for fungi. The Specaccum curves based on OTU number (Supplementary Figure 2), approaching the saturation plateau, suggested that the sequencing depth in this research was adequate to represent the microbial structure of samples. We determined the diversity of bacterial and fungal communities with standardized sequences. The characteristics of microbial communities are listed in Table 1 (α Diversity index). According to the Shannon index, the bacterial diversity of Daqu in fermentation first decreased, then increased. Besides, the Simpson index showed an identical trend in the fermentation process.

**TABLE 1:** α-diversity indices for microbial communities among samples.

		**Organic acids in Daqu samples during fermentation**						
**Samples**	**Oxalic acid mg/g**	**Tartaric acid mg/g**	**Malic acid mg/g**	**Lactic acid mg/g**	**Acetic acid mg/g**	**Citric acid mg/g**	**Succinic acid mg/g**	**Fumaric acid mg/g**
D0	0.064856061	1.376650975	0.17305526	2.898827634	2.362910505	0.123592621	0.150312897	0.016473083
D6	0.297644626	0.558619726	0.572926375	3.268853984	1.955273719	0.29636085	10.66781341	0.130289244
D16	0.20937223	0.67371888	0.565720883	1.345282419	4.050033926	0	14.77240691	0.068486009
D29	0.166994506	0.626178531	0	0.631942795	8.084225285	0	0	0.087137844

**FIGURE 2:**
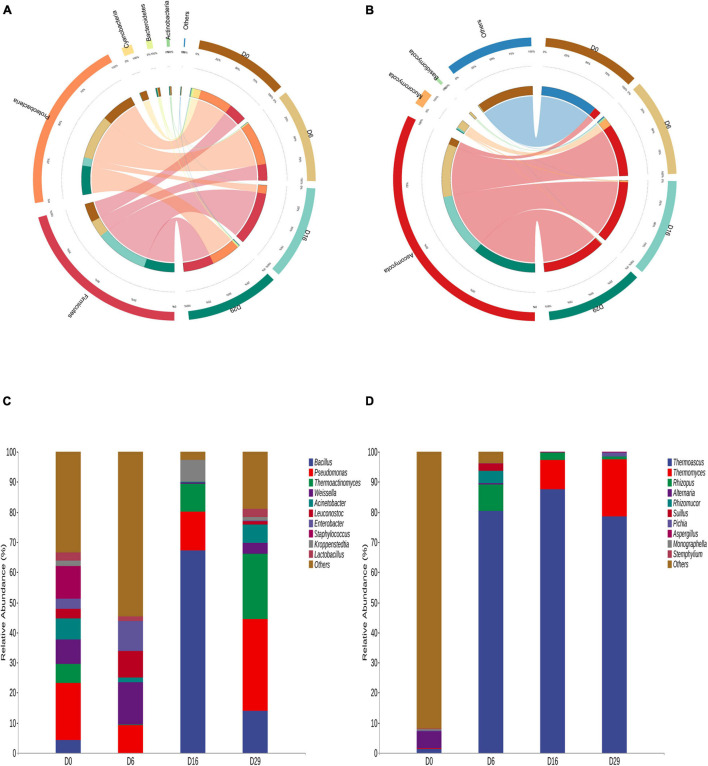
Distribution of bacteria **(A)** and the fungi **(B)** on phylum level during the fermentation process of Daqu. And showing the changes of major bacteria **(C)** and fungi **(D)** in the samples during the fermentation on genus level.

The differences in microbial communities among the four *Daqu* samples became very obvious by using Circos (Ye et al., 2014) on the phylum level. Firmicutes, Proteobacteria, Cyanobacteria, Bacteroidetes and Actinobacteria were the main bacterial phyla (Figure 2A). Ascomycota, Mucoromycota and Basidiomycota were the predominant fungal phylum, comprising more than 98.68% of the total fungal population except for 0 day (Figure 2B). Meanwhile, the dissimilarities in genera in the four samples were apparent (Figures 2C,D). *Bacillus*, *Thermoatinomyces*, *Pseudomonas*, and *Weissella* were the main bacterial genera. However, the dominant bacteria genus were different in the four fermentation time samples. Initially, the relative abundance of *Pseudomonas, Weissella and Staphylococcus was* 37.86% in total. On the sixth day, the relative abundance of *Weissella* was 13.98%. On the 16 th day, the relative abundance of *Bacillus* was 67.34%, On the 29 th day, the relative abundance of *Pseudomonas* and *Thermoatinomyces* was 51.98% in total. In addition, *Weissella*, *Leuconostoc*, and *Lactobacillus* were the main Lactic acid bacteria in the fermentation process at four time points. The fungi community was dominated by *Thermoascus*, *Thermomyces*, *Rhizopus*, and *Alternaria* during the 29-day fermentation of Daqu. *Thermoascus* is the most dominant genus (more than 78.56%) except for 0 day. Nevertheless, the dominant genera were different in the four fermentation time samples. On the sixth day, the fungal community was dominated by *Thermoascus* (80.40%) and *Rhizopus* (8.79%). On the 16 th day, the main geuns were *Thermoascus* (87.53%) and *Thermomyces* (9.71%). Identically, On the 29 th day, the dominant genus were either *Thermoascus* (78.56%) and *Thermomyces* (19.01%). In addition, in unfermented Daqu samples, the most principal fungi in the microbial community were some unclassified fungi. This may be some unique microorganisms in the raw material.

### Comparison of Microbial Community Composition

**FIGURE 3:**
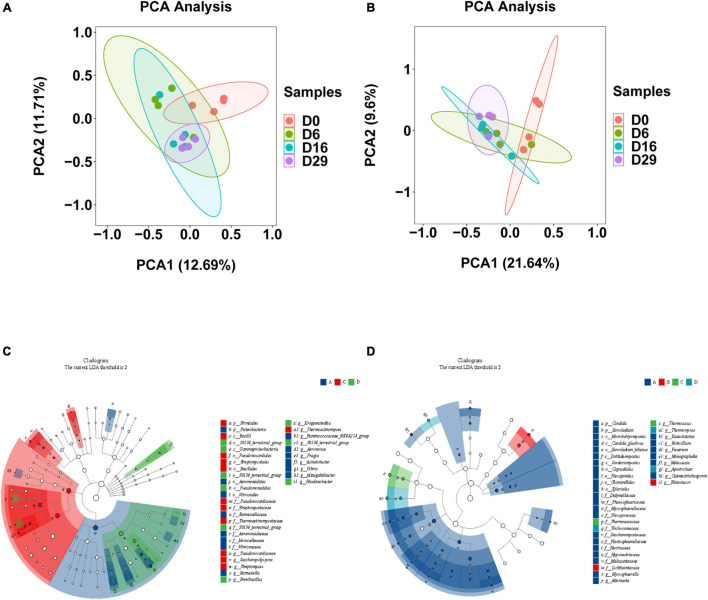
Clustering analysis of bacteria **(A)** and the fungi **(B)** in different fermented samples (days 0, 6, 16 and 29)using principal component analysis. The differential bacteria **(C)** and fungi **(D)** in each time point of Daqu by using LEfSe analysis (LDA > 2, *p* < 0.05) (Four groups were set according to four time points).

The significance of the difference of bacterial and fungal communities in samples from different fermentation stages is shown as a principal component analysis (PCA) plot (Figures 3A,B). However, roughly, the differences between them are not obvious. This may be because Daqu fermentation is a complex system in which microorganisms interacted with each other. We used LEfSe analysis to calculate in addition to the main differential microorganisms in each group. In total, 17 bacterial genus and 17 fungi genus showed significant differences among samples (Figures 3C,D, LDA > 2, *p* < 0.05). In the beginning, the abundance of *Aeromonas*, *pragia*, *Acinetobacter*, and *Mizugakiibacter* was significantly higher than those at other time points. Finally, the abundance of *Acinetobacter* decreased remarkably, while *Thermoactinomyces*, *Kroppenstedtia*, and *BreviBacillus* were the dominant bacteria, which accounted for 67.62% of the total bacteria. In the early stage, *Mycosphaerella*, *Alternaria*, *Kazachstania*, *Verticillium*, and *Fusarium* were the main fungal genera, while the abundance of *Mycosphaerella*, *Alternaria*, and *Kazachstania* was significantly decreased. On the sixth day, *Rhizomucor* (81.32% of the total fungi) was the dominant fungal genus. In the later stage, *Thermoascus* (14.87%), *Thermomyces*, and *Pichia* (61.10%) were the main genera of the fungal community.

### Prediction of Functional Genes in the Daqu Microbial Community

**FIGURE 4:**
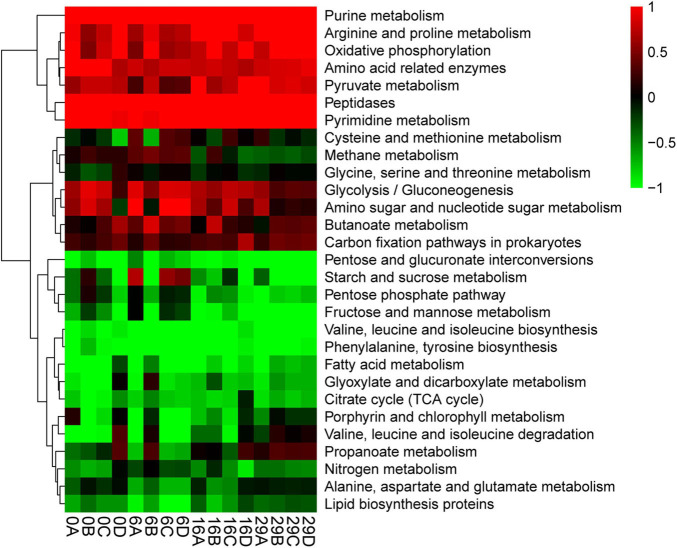
Potential function prediction (based on the level-3 pathway in metabolite of KEGG database).

The analysis using the PICRUSt2 software package compared the functional characteristics of the microbial community in Daqu (He et al., 2020). The accuracy of the functional prediction using PICRUSt2 is reported so it could reach 85%–90% for microorganisms. The PICRUSt2 software package. Make metabolism as the level-1 pathway and 115 level-3 pathways are identified in the KEGG pathways. We screened the pathways of the top 30 abundances and made a heatmap (Figure 4). Generally, the relative rate of pentose and glucuronate interconversions was the highest. Especially at the beginning and end of fermentation. The relative abundances of starch and sucrose metabolism gradually increased during the fermentation as well as Glycolysis/Gluconeogenesis. The relative abundances of Glycolysis/Gluconeogenesis were low at the beginning of fermentation. The relative abundances of Glyoxylate and Dicarboxylate metabolism have a high relative abundance, which increased at the beginning of fermentation and reduced gradually, whereas, the relative abundances of the Citrate cycle (TCA cycle) and Lipid biosynthesis first increase and then decrease. It is noteworthy that not only the relative abundances of phenylalanine and Tyrosine biosynthesis gradually increase during the fermentation of Daqu; but also in the whole process, its abundance is at a high level.

### Metabolomics Analysis of Fermented Daqu

**FIGURE 5:**
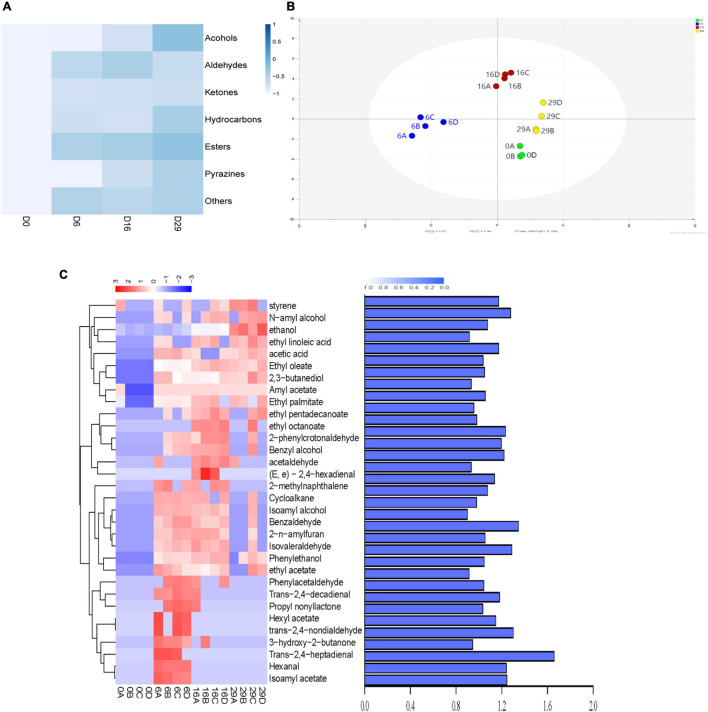
Kinetic comparison of volatile metabolites during fermentation. **(A)** Heatmap showing the Changes of different types of compounds in the fermentation process. **(B)** Clustering analysis of the volatile compounds in different fermented samples by orthogonal partial least squares analysis. **(C)** Distribution of different metabolites and value of variable importance in metabolite projection.

In non targeted metabonomics analysis (based on GC-MS), 102 volatile compounds were found in the fermentation process. These compounds were divided into esters, alcohols, aldehydes, hydrocarbons, pyrazines, ketones, and other heterocyclic compounds (Figure 5A). Among them, 35 esters accounted for the highest proportion, followed by 22 aldehydes. During the whole fermentation process, the total volatile content of Daqu increased to 0.6882 mg/kg, which was about 5 times that of non fermented Daqu. Esters were the dominant volatile substances during the fermentation process of Daqu; their contents reached 0.0706 mg/kg on day 29 of the fermentation process. The ester content increased from 0.0129 mg/kg in the unfermented Sample to 0.0706 mg/kg on day 29 of the fermentation process. The scatter diagram (using OPLS-DA analysis) shows the samples with different fermentation times according to the concentration and type of each substance (Figure 5B). Differential metabolites (DMs) were are defined as the product with variable importance (VIP) value of ≥ 1 in the prediction and *P* value of ≤ 0.05 in the *t*-test. Figure 5C shows that there were 32 DMs among the samples and their scores of VIP, including 10 Esters, 6 Hydrocarbons, 10 Aldehydes, and 6 Alcohols. Among these, 16 Volatile metabolites were detected, which contributed to flavor characteristics. During the fermentation of Daqu, the concentrations of most of the esters and the alcohols significantly increased after fermentation, such as those of Amyl acetate (0 to 0.0113 mg/kg), Ethyl octanoate (0 to 0.0009 mg/kg), Ethyl pentadecanoate (0 to 0.0004 mg/kg), Ethyl palmitate (0 to 0.0224 mg/kg), Ethanol (0.0086 to 0.0737 mg/kg), N-amyl alcohol (0 to 0.0028 mg/kg), and phenylethanol (0 to 0.0047 mg/kg).

### Changes in Organic Acids During Fermentation

During the fermentation process, the change of organic acids is an important physical and chemical index that can reflect the important activity of microorganisms indirectly. Table 2 shows that the total content of organic acids increased from 7.1667 mg/g to 9.5964 mg/g. In the fermentation of Daqu, Acetic acid was the most important organic acid, accounting for 84.24% of the total organic acids on the 29 th day of fermentation. After 29 days of fermentation, its content increased from 2.3629 mg/g to 8.0842 mg/g. However, with time, the content of lactic acid and fumaric acid increased first and then decreased. Their content both reached the highest point on the sixth day at 3.2688 mg/g, 0.1302 mg/g respectively. Then the content of lactic acid began to decrease significantly and reached 0.6319 mg/g at the end of fermentation. In addition, the content of citric acid was always at a low level, only 0.2963 mg/g at most (The sixth day of fermentation), and could not be detected after 16 days of fermentation.

**TABLE 2:** Contents of organic acids in Daqu samples.

**Samples**	**Abundance indices**				**Diversity indices**			
	**Chao1**		**ACE**		**Simpson**		**Shannon**	
	**Bacteria**	**Fungi**	**Bacteria**	**Fungi**	**Bacteria**	**Fungi**	**Bacteria**	**Fungi**
D0	878.76	104.17	920.73	104.5	0.944182	0.856625	5.83	3.56
D6	545.88	216.78	557	216.23	0.776769	0.850502	3.94	1.77
D16	215.37	83.06	222.8	87.21	0.789673	0.252791	3.65	0.84
D29	603.8	96.65	611.67	100.88	0.913594	0.384877	5.25	1.17

### The Correlation Between Differential Microbial Communities and DMs

**FIGURE 6:**
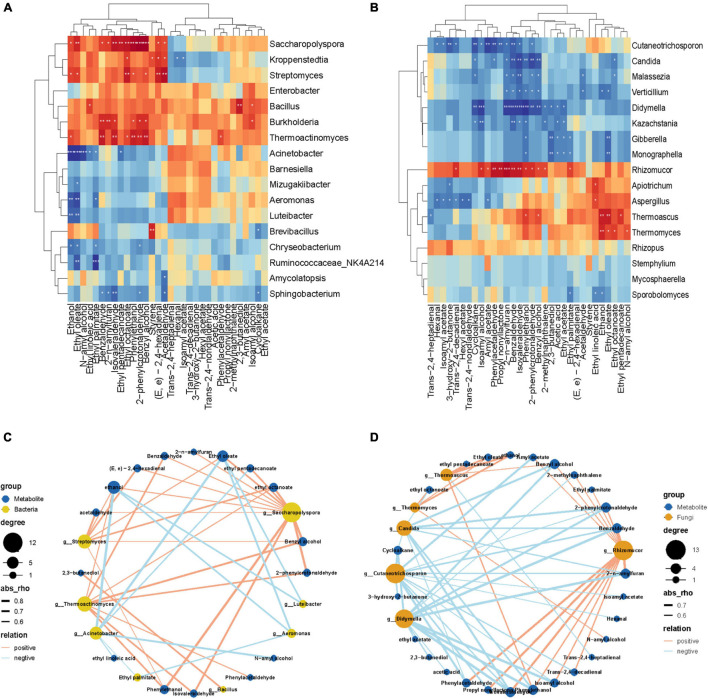
Heatmaps of Spearman correlations between dominant **(A)** bacterial and **(B)** fungal genera and differential metabolites during the fermentation process. Levels of significance are shown as follows: *0.01 < *p* ≤ 0.05; **0.001 < *p* ≤ 0.01,***p ≤ 0.001. The co-occurrence network diagram of a highly significant correlation between differential metabolites and differential bacteria **(C)** and fungi **(D)**.

The correlation between different microorganisms and DMS in the fermentation process was analyzed. In the key bacterial communities (Figure 6A), 17 microbes exhibited an association with DMs (| *ρ*| > 0.6). Only eight bacteria have a significant correlation with DMs (*p* < 0.005). As Figure 6C shows, the abundance of *Streptomyces* has a very significant positive correlation with Acetaldehyde, Ethyl octanoate, (*E*, *e*)-2,4-hexadienal, Ethyl oleate, Ethanol, Benzyl alcohol, and Phenylethanol. In addition, the abundance of *Bacillus* was positively correlated with only 2,3-butanediol. However, the abundance of *Acinetobacter* had a significantly negative correlation with Ethanol, Ethyl oleate, *N*-amyl alcohol, Ethyl palmitate, and Ethyl linoleic acid. It is noteworthy that the abundance of *Saccharopolyspora*, *Streptomyces*, and *Thermoactinomyces* have a simultaneously extremely significantly positive correlation with Ethyl octanoate, Phenylethanol, and Benzyl alcohol. Whereas, the abundance of *Acinetobacter*, *Aeromonas*, *Luteibacter* and *Chryseobacterium* positively correlated with ethanol and Ethyl oleate contents.

In the key fungal communities (Figure 6B), 17 microbes exhibited a high association with DMs (| *ρ*| > 0.6)). In fungi genera, *Rhizomucor* was positively correlated with 12 DMs, including *Trans*-2,4-decadienal, Iso-amyl alcohol, Amyl acetate, Phenylacetaldehyde, Propyl nonyllactone, 2-*n*-amylfuran, Benzaldehyde, Iso-valeraldehyde, Phenylethanol 2-Phenylcrotonaldehyde, Benzyl alcohol, 2-methylnaphthalene, and Ethyl palmitate. However, there was a significant positive correlation between *Cutaneotrichosporon* and 13 DMs, containing Hexanal, Isoamyl acetate, 3-hydroxy-2-butanone, T*rans*-2,4-decadienal, Cycloalkane, Isoamyl alcohol, Amyl acetate, Phenylacetaldehyde, Propyl nonyllactone, 2-*n*-amylfuran, Benzaldehyde, Isovaleraldehyde and 2-phenylcrotonaldehyde. As we can see from the correlation network (Figure 6D) diagram, which shows that the abundance of *Candida* and *Didymella* have a simultaneous extremely significant negative correlation with the contents of 2-*n*-amylfuran, Benzaldehyde, Isovaleraldehyde, Phenylethanol, 2-phenylcrotonaldehyde, and Benzyl alcohol. In the meantime, the abundance of *Thermoascus* and *Thermomyces* showed a significantly positive correlation with ethanol and ethyl oleate contents.

### Pathway Enrichment Analysis

**FIGURE 7:**
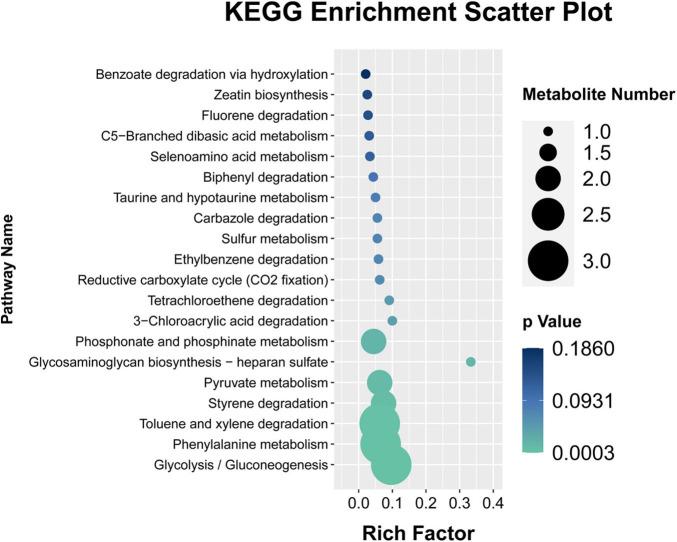
KEGG pathway enrichment of identified differential metabolites.

The KEGG database was analyzed to enrich the pathways of DMs (Xu et al., 2021). To further explore the differential expression of these aforementioned metabolites in the fermentation process. A total of 21 DMs were identified and measured, and their contents determined. Metabolites set enrichment pathways analysis displayed that there are 19 pathways in all, there are six pathways of significant enrichment (Figure 7). They are Glycolysis/Gluconeogenesis, Phenylalanine metabolism, Toluene and xylene degradation, Styrene degradation, Pyruvate metabolism, Glycosaminoglycan biosynthesis–heparan sulfate, Phosphonate and Phosphinate metabolism, 3-Chloroacrylic acid degradation, Tetrachloroethene degradation (*p* < 0.05). Glycolysis/Gluconeogenesis and Phenylalanine metabolism are highly enriched pathways, with a *p* value of 0.0003 and 0.0009, respectively. At the same time, these two pathways also contain the largest number of DMs. Interestingly, these two pathways are also highly significant in the previous PICRUSt2 function prediction results of different microbial communities.

## Discussion

This study aimed to investigate the relationship between metabolites and microbial community changes by using a combination of microbiomics and metabolomics, to provide a theoretical basis for the quality monitoring of Daqu fermentation (Zheng et al., 2011). We determined the metabolites of Daqu during the fermentation and then undertook a cluster analysis (OPLS-DA) that showed that there were significant differences on days 0, 6, 16, and 29. We found that this difference was similar to the succession of microbial communities during fermentation by sequencing the amplicon of the sample. The amplification and sequencing of 16S rRNA and ITS gene showed that the microbial community was mainly composed of three prokaryotic genera (*Bacillus*, *Pseudomonas*, and *Enterobacter*) and three eukaryotic genera (*Aspergillus*, *Rhizopus*, and *Thermoascus*), which are identical to many previous reports.

The key microorganisms in the samples of four fermentation time points were selected through LEfSe analysis. The early bacteria were mainly *Aeromonas*, *pragia*, *Acinetobacter*, and *Mizugakiibacter*. The late bacteria were mainly *Thermoactinomyces*, *Saccharopolyspora*, *Streptomyces*, and *BreviBacillus.* Whereas at the early stage, *Mycosphaerella*, *Alternaria*, *Kazachstania*, *Verticillium*, and *Fusarium* were the main genus of fungi. On the sixth day, *Rhizomucor* was the dominant fungal genus. We found that it showed significant positive correlations with five kinds of DMs (Isovaleraldehyde, Benzaldehyde, 2-n-amylfuran, Propyl nonyllactone, Phenylacetaldehyde), In previous studies, *Rhizomuco* was a popular genera in Daqu and are considered to be important functional micro organizations. As a powerful amylase producer, *Rhizomucor* is widely used in rice wine brewing and other traditional fermented foods by decomposing starch in Daqu raw materials, promoting the transformation of Pyruvic acid and Amino acid, providing nutrients for the growth of other microorganisms, and accumulating reducing sugar for later alcohol fermentation (Dung et al., 2007; Jiang et al., 2020). Additionally, the thermal fungus *Rhizomucor* has been used for producing industrial proteins and lipids (Yang et al., 2015; Wang et al., 2020). *Rhizomucor* can also secrete lipases and proteases, which play a key role in the process of synthesis of volatile substances, such as Esters, Aldehydes, Fatty acids, and so on. This explains that there are significant positive correlations between *Rhizomucor* and various DMs. Therefore, we can detect the concentration of changes of Isovaleraldehyde, Benzaldehyde, Phenylacetaldehyde, and other substances in the early stage to reflect the abundance changes of *Rhizomucor* in the early stage of fermentation in Nongxiang Daqu.

At the later stage, *Thermoascus* and *Thermomyces* were the differential genus of the fungal community. A higher abundance of *Bacillus* was observed in the 16 th to 29 th days of fermentation. First, this could be because the oxygen content is sufficient in the early stage of fermentation. The rapid growth of some aerobic microorganisms in Daqu had adverse effects on Bacillus. Second, Bacillus may compete with other bacteria for common ecological fields (Prosser et al., 2007). Third, Some bacteria may be inhibited by fungi, which reduce pH or produce ethanol. For example, *S. cerevisiae* can inhibit the growth of other strains by ethanol production (Meng et al., 2015). While for some yeasts, the growth of *Pichia* is inhibited by *Bacillus* (Deng et al., 2020). In the middle and later stages of this experiment, *Bacillus* dominated the microbial community. This is the same as the previous conclusion. *Bacillus* is a principal producer of Daqu flavor. It can secrete various hydrolases, including amylase, protease, and lipase to hydrolyze macromolecules, and produce flavor compounds in the brewing process (Zheng et al., 2011). More importantly, the production of hydrolytic enzymes and flavor precursors was improved after co-culture with *Bacillus*. The corresponding samples also exhibited higher contents of aromatic compounds, including, (2,2-diethoxyethyl)-benzene, Benzeneethanol, and Ethyl phenylacetate. Especially for Benzeneethanol and ethyl, they could provide rose-like and honey fragrances for liquor (Fan et al., 2007; Wang P. et al., 2017). We then found that there was only one significant positive correlation between *Bacillus* and 2,3-butanediol production, 2,3-Butanediol, a normal constituent of wine, is one of the rare aroma-producing polyols (Minebois et al., 2020). It might be synthesized from pyruvate by *Bacillus* (Wu et al., 2014). Furthermore, *Bacillus*, as a secretor of amylase, can continuously convert fermentable substances into Pyruvate. It may also be derived from diacetyl and acetoin in *Leuconostoc* (Escobar-Zepeda et al., 2016), but according to the microbial data of the Daqu fermentation process it can be observed that the metabolic activity of Leuconostoc is not evident. Therefore, 2,3-butanediol may be one of the biomarkers reflecting the growth of *Bacillus*.

In addition, the relative abundance of *Thermoactinomyces* increased significantly in the later stage of fermentation. These organisms are known to survive in harsh environments and metabolize at 45°C to produce various thermostable enzymes, such as amylase, serine, protease, and lipase (Pan et al., 2016). The existence of these bacteria can effectively promote the aerobic fermentation process of Daqu, which is consistent with the above results. During the aerobic fermentation of Daqu, the active metabolites of thermostable enzymes remained at a high level. Therefore, in the late stage of fermentation, we can determine that the relative abundance of *Thermoactinomyces* in Daqu samples could be determined by monitoring the contents of these metabolites. In addition, in the early stage of fermentation, *Candida* was selected as a differential microorganism. *Candida* is one of the producers of esters and alcohols (Han et al., 2009). It is the main microorganism responsible for lactic acid fermentation and alcohol fermentation (Sakandar et al., 2020). Ester production occurs in the downstream part of glycometabolism. *Candida*, *Aspergillus*, *Rhizopus* (Gandolfi et al., 2001) and *Lactobacillales* (*Enterococcus*, *pediococcus*, and *LactoBacillus*) participated in the production of esterase. In the amino acid branch, phenylethyl alcohol was produced from phenylalanine metabolism through the Ehrlich pathway of yeast. *Candida* and *Monascus* (Rahayu et al., 2017) participate in this process. In this study, we observed a significant positive correlation was observed between *Aspergillus* and ethyl linoleic acid. Previous studies have found that unsaturated fatty acids including linoleic acid, unsaturated fatty acid − rich fraction could be liberated by the action of *Aspergillus oryzae* lipases (Sakandar et al., 2020). It is noteworthy that Ethyl linoleate does not exist in Daqu, but appears in the middle and late stages of the fermentation process. Therefore, The increase of fatty acid ethyl ester content is obviously due to lipase catalyzed ester conversion as reported by Mitsuaki (1996), which indicated out that Fatty acid ethyl ester can be produced in fermented soybean paste when ethanol content reached 0.1% or more. In our case, the content of this ester was zero at the beginning, probably because yeast and Lactobacillus had no active metabolic activity, but its obvious formation result was produced a few days after. This shows that the enzyme reaction was effectively carried out in the fermentation process. This process is particularly crucial because fatty acid esters directly or indirectly promote the ripening flavor of any fermented food. It may thus be possible to follow up the fermentation process by monitoring the increase of ethyl linoleic acid content.

Previous research has suggested that the fermentation environment, microbial populations, and original raw materials notably influence the content of the organic acid. Acetic acid is the main organic acid at the end of Daqu fermentation, which is 8.0842 mg/g. On the one hand, this may be due to bacteria, on the other hand, it is the main end product in the high abundance pathways screened by PICRUSt2 function prediction and the pathway enrichment analysis of DMs. At the same time, as the intermediate product of some metabolic pathways, a part of Acetic acid will be metabolized by microorganisms in some communities (Nie et al., 2013), which could explain the phenomenon that the content of Acetic acid increases significantly and then decreases gradually during the fermentation process. In addition, most *LactoBacillus* species were capable of producing Lactic acid and Acetic acid (Rubing et al., 2020), The major citric acid accumulating microorganisms include several yeast strains from the genera *Aspergillus* and *Candida*, whereas the major malic acid producing microorganisms are from the genera *Saccharomyces* and *Aspergillus* (Ren et al., 2019). *Staphylococcus* and *LactoBacillus* could produce Lactic acid, and *Saccharopolyspora* might utilize Lactic acid, while *Saccharomyces* and *Aspergillus* were involved in both the production and utilization of Lactic acid (Liu et al., 2019). The interplay between these organisms was likely the reason for the fluctuation of the concentration of Lactic acid during the fermentation process, which may indicate that the determination of organic acid. content and its change can also reflect the growth and abundance of microbial flora related to organic acid production in the Daqu sample.

**FIGURE 8:**
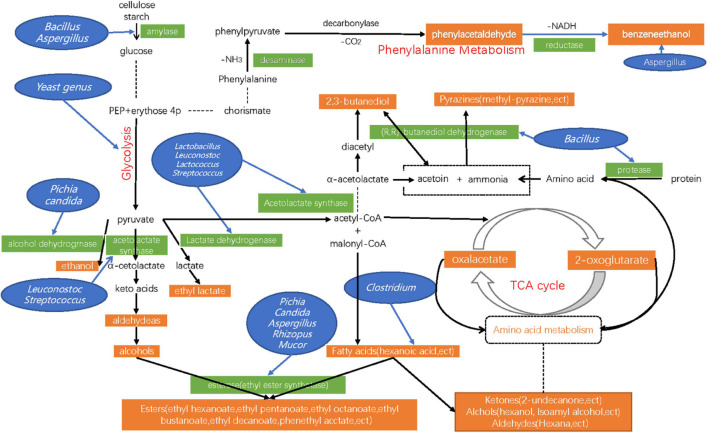
Schematic presentation of pathways, metabolites, enzymes, and genus related to the synthesis of Ethanol, Phenylethanol, Phenylacetaldehyde, and Lactic acids, ect in Daqu samples. The pathways were organized based on the KEGG (Kyoto Encyclopedia of Genes and Genomes).

An integrated pathway map was used to show the metabolic pathways of some DMs in detail (Figure 8). Among the multiple pathways, Glycolysis/Gluconeogenesis pathway, Phenylalanine metabolism pathway, Pyruvate metabolic pathway, and TCA Cycle are directly involved in the production of Acetate, Acetaldehyde, Ethanol, Phenylacetaldehyde, and Phenylethyl alcohol (Jin et al., 2019; Liu et al., 2019). In the phenylalanine metabolism pathway, the levels of three proteins (Acetyl-CoA, Succinyl-CoA, and Phenyl-acetyl-CoA) and two metabolites (Phenylacetaldehyde, and Phenylethyl alcohol) were enriched. This result is consistent with the change of Phenylacetaldehyde and Phenylethyl alcohol contents in the previous volatile metabolite test during the fermentation of Daqu. Previous studies showed that the amylase and protease secreted by Bacillus changed starch and protein into glucose and amino acids, and promoted the formation of flavor compound precursors during the fermentation of Baijiu (Huang et al., 2020). They then also participated in the synthesis of a variety of volatile metabolites (Wang X. et al., 2017), However, many other microorganisms, such as some Lactic acid bacteria and thermophilic fungi also play this role (Jin et al., 2019; Yang et al., 2021). Therefore, we need to use more accurate omics sequencing and analysis technology to determine which microorganisms participate in specific metabolic pathways and which links are involved in different fermentation periods. It is a complex process, especially in Daqu fermentation. In such an open environment, the metabolic activities of various microorganisms form a very complex metabolic network. We still need to improve the understanding of flavor-producing core microorganisms.

## Conclusion

In this study, we explored the correlation between the dominant microorganisms and different metabolites in different stages of the fermentation process, and then verified the relationship by using functional prediction and pathway enrichment. The result showed that the dynamic changes of microbial community were significantly correlated with the changes of some metabolites during the fermentation of Nongxiang Daqu. Thermoascus is the main fungus genus during the whole fermentation process. Its relative abundance was positively correlated with Ethanol and Ethyl oleate content. Meanwhile, in the middle and late stages of fermentation, the bacillus is the main bacteria. In addition, the change in the content of 2,3-butanediol was positively correlated with the change in the abundance of Bacillus, which can provide a new perspective for further exploring the relationship between microorganisms and metabolites in Daqu fermentation. Studying the changes of metabolites to reflect the changes of microorganisms in the fermentation process could provide a way to realize the rapid detection of Daqu quality strains and help control the microorganisms in the Daqu making process, to obtain more stable Baijiu products.

## Data Availability Statement

The original contributions presented in the study are publicly available. This data can be found here: National Center for Biotechnology Information (NCBI) BioProject database under accession number PRJNA718914.

## Author Contributions

HL, DH, and BH provided the idea, the framework, and support for the research. DY, YZ, HG, and WJ participated in the experiment. YD generated the data and performed the analysis of bioinformatics. YD wrote and edited the manuscript. All the authors contributed to the article and approved the submitted version.

## Conflict of Interest

DY, HG, YZ, and WJ were employed by company Sichuan Tuopai Shede Liquor Co., Ltd. The remaining authors declare that the research was conducted in the absence of any commercial or financial relationships that could be construed as a potential conflict of interest.

## Publisher’s Note

All claims expressed in this article are solely those of the authors and do not necessarily represent those of their affiliated organizations, or those of the publisher, the editors and the reviewers. Any product that may be evaluated in this article, or claim that may be made by its manufacturer, is not guaranteed or endorsed by the publisher.

## Footnotes

1
http://ccb.jhu.edu/software/FLASH/

2
http://www.genome.jp/kegg/pathway.html
