# Supplementary figures and images for: Correlation: Between Autochthonous Microbial Diversity and Volatile Metabolites During the Fermentation of Nongxiang Daqu
Source: Front Microbiol. 2021 Sep 22;12:688981. doi: 10.3389/fmicb.2021.688981 (PMC8494108; doi:10.3389/fmicb.2021.688981)

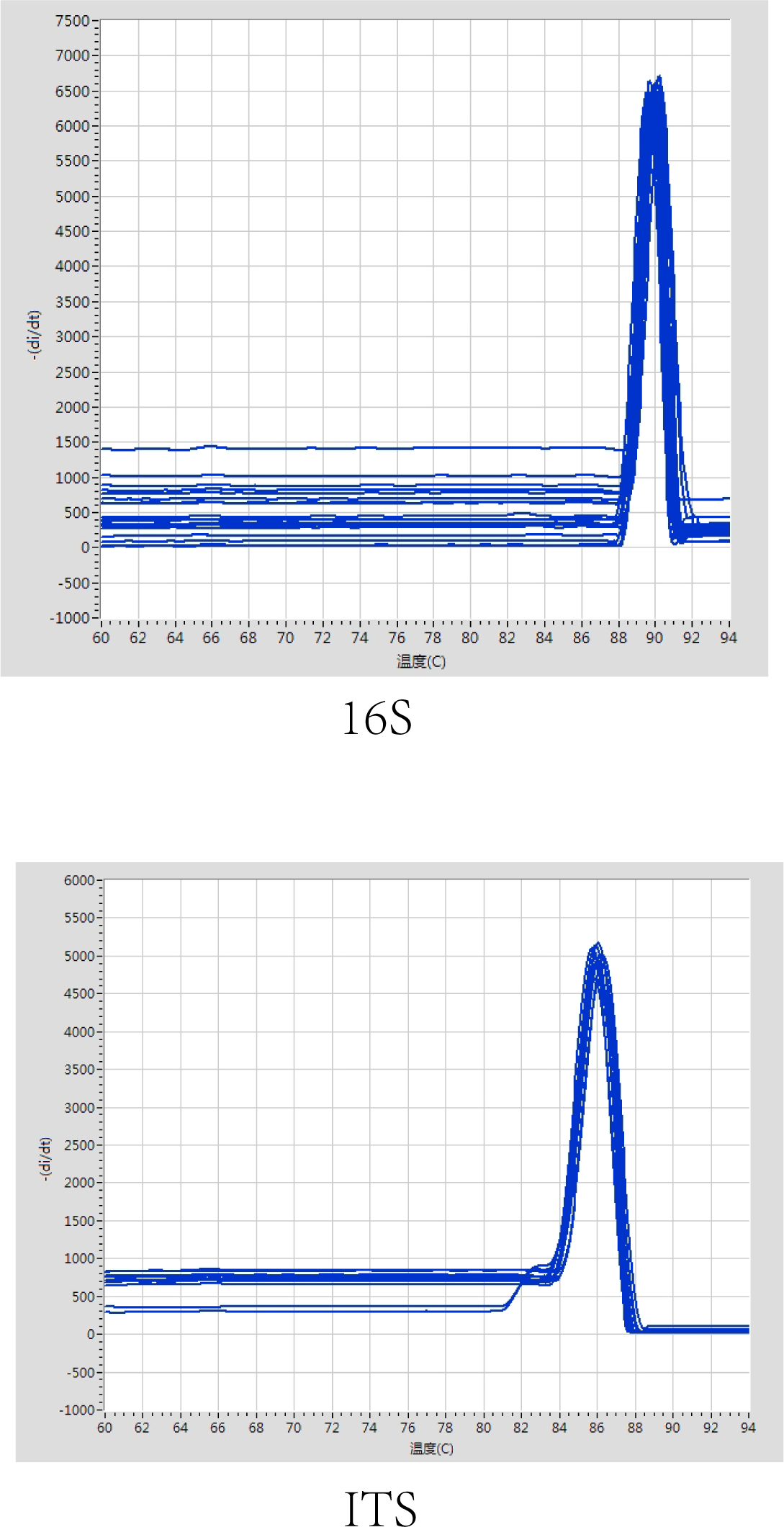

Supplement: Supplementary file 6 [file Image_1.tif]

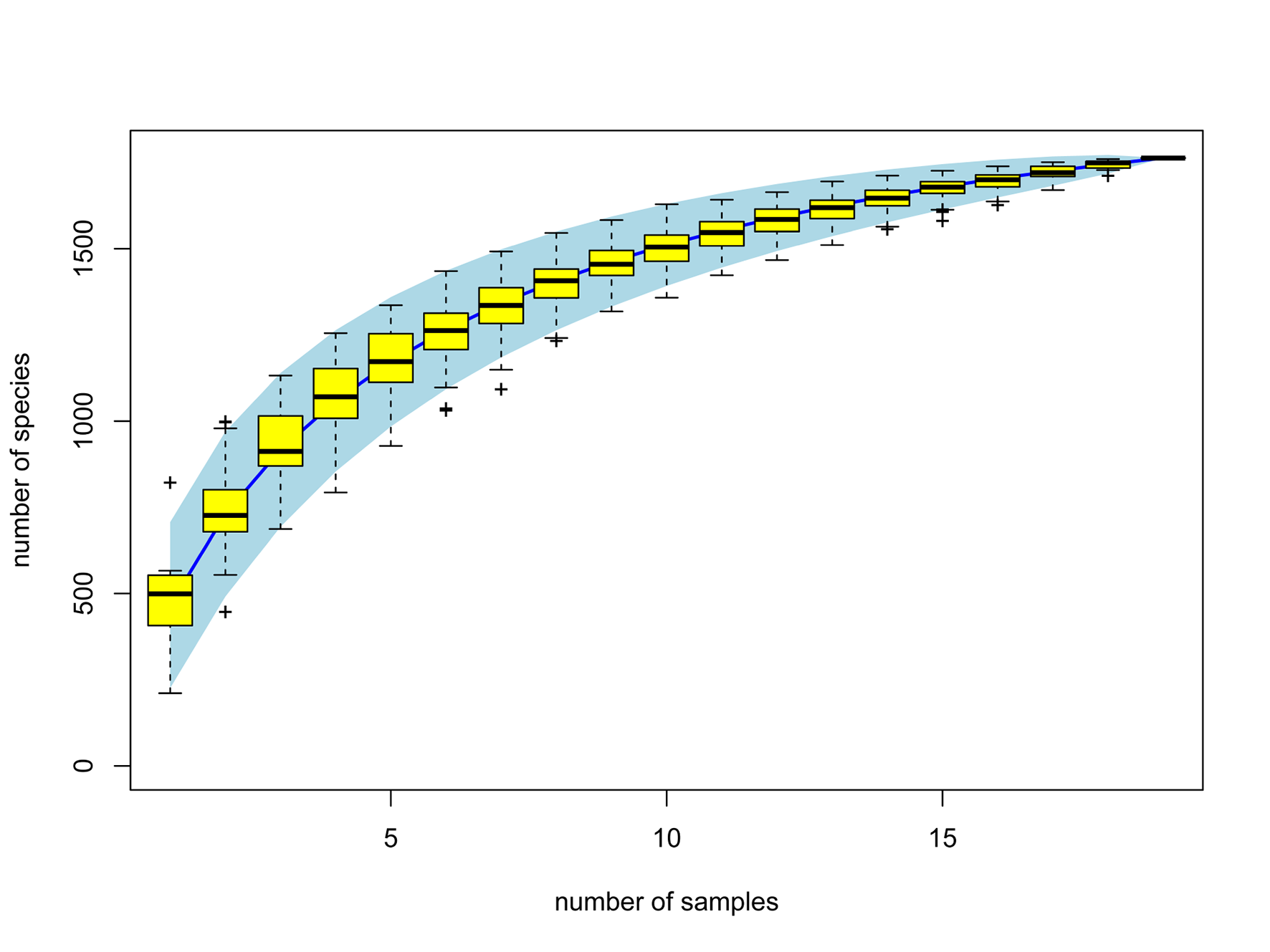

Supplement: Supplementary file 7 [file Image_2.tif]
